# Supplementary material for: 4-Amino-TEMPO-Immobilized Polymer Monolith: Preparations, and Recycling Performance of Catalyst for Alcohol Oxidation
Source: Polymers (Basel). 2022 Nov 24;14(23):5123. doi: 10.3390/polym14235123 (PMC9737294; doi:10.3390/polym14235123)
Supplement: Supplementary file 1 [file polymers-14-05123-s001.zip › polymers-2002806-supplementary.pdf]

Supporting Information

4-Amino-TEMPO-Immobilized Polymer Monolith: Preparations, and Recycling Performance of Catalyst for Alcohol Oxidation

1. Poly (4-chloromethyl-*co*-divinylbenzene) (Cl−Monolith)

Table S1. Properties of Cl−Monolith−1, −2, −3, and −4 as characterized by MIP.

Material	DVB [wt %]	Specific Surface Area [$m^2 g^{-1}$]	Average Pore Diameter [μm]	Median Pore Diameter [μm]	Porosity [%]
Cl−Monolith−1	20	1.1	4.8	17	58
Cl−Monolith−2	30	3.8	1.9	12	63
Cl−Monolith−3	40	27	0.24	12	65
Cl−Monolith−4	50	47	0.15	1.7	66

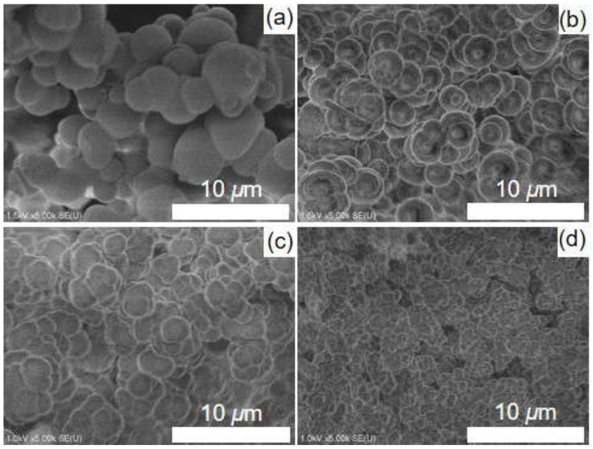

Figure S1. SEM images of Cl−Monolith (a) −1, (b) −2, (c) −3, and (d) −4.

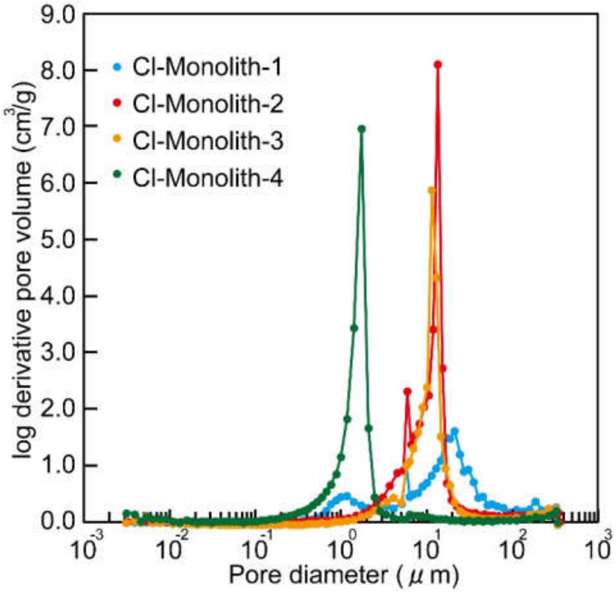

Figure S2. Pore size distribution of Cl−Monolith−1, −2, −3, and −4 as characterized by MIP.

Table S2. The transmission coefficient of Cl−Monolith−1, −2, −3, and −4.

Material	DVB [wt %]	Trasmission Coefficient [m^2][a]
Cl−Monolith−1	20	8.3×10^{-12}
Cl−Monolith−2	30	3.1×10^{-13}
Cl−Monolith−3	40	7.0×10^{-14}
Cl−Monolith−4	50	3.7×10^{-14}

[a] calculated by Darcy's law.

2. 4-Amino-TEMPO-immobilized monolith (Monolith)

Table S3. Properties of Monolith−1, −2, −3, and −4 as characterized by MIP.

Material	DVB [wt %]	Specific Surface Area [m^2 g^{-1}]	Average Pore Diameter [μm]	Median Pore Diameter [μm]	Porosity [%]
Monolith−1	20	0.85	8.8	14	65
Monolith−2	30	0.74	8.4	13	61
Monolith−3	40	18	0.34	4.3	59
Monolith−4	50	29	0.23	0.57	64

Table S4. N content and 4-amino-TEMPO immobilization calculated from elemental analysis of Monoliths, and transmission coefficient.

Material	DVB [wt %]	N [mmol/g][a]	4-amino-TEMPO [mmol/g][a]	Trasmission Coefficient [m^2][b]
Monolith−1	20	1.1	0.53	5.7×10^{-12}
Monolith−2	30	0.73	0.36	4.1×10^{-13}
Monolith−3	40	0.66	0.33	7.3×10^{-14}
Monolith−4	50	0.49	0.24	3.5×10^{-14}

[a] calculated by elemental analysis, [b] calculated by Darcy's law.

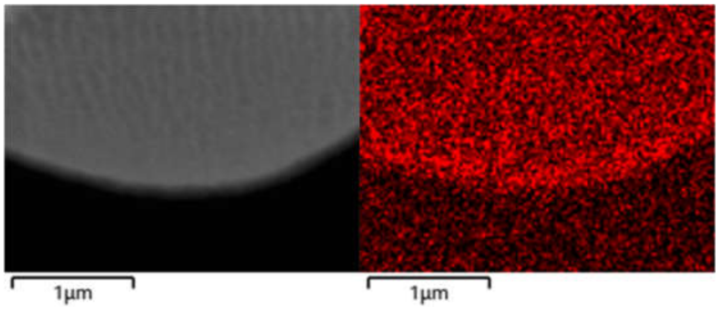

Figure S3. SEM image (left) EDS image analyzing nitrogen (right) of Monolith−1.

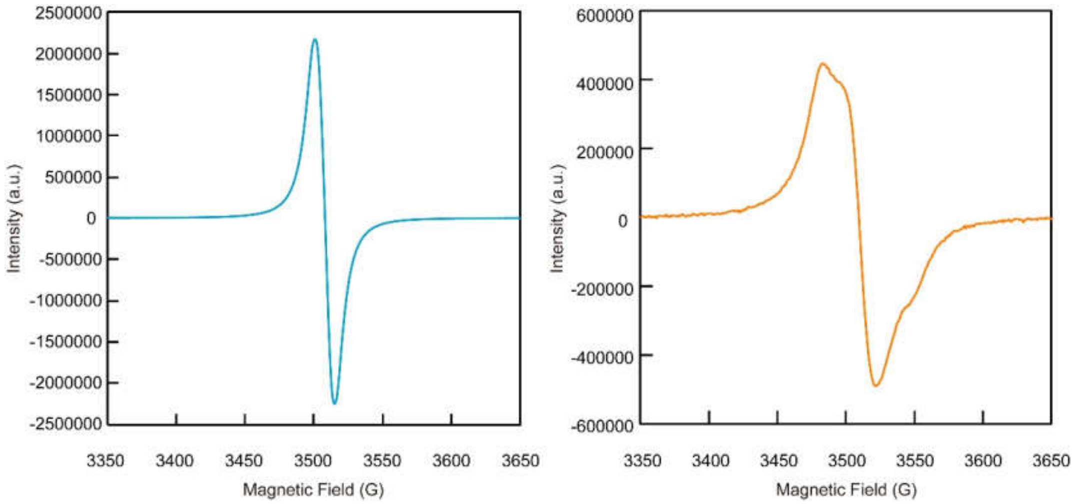

Figure S4. The ESR spectra of TEMPO (5 mg, left) and Monolith−1 (8 mg, right). The amount of 4-amino-TEMPO immobilized in Monolith−1, hypothetically calculated from two integrations of ESR spectra ranged from 0.43–0.69 mmol/g. (Calibration curves were prepared from ESR spectra of different amounts of TEMPO.) This value contains uncertainty due to the influence of radical interactions.

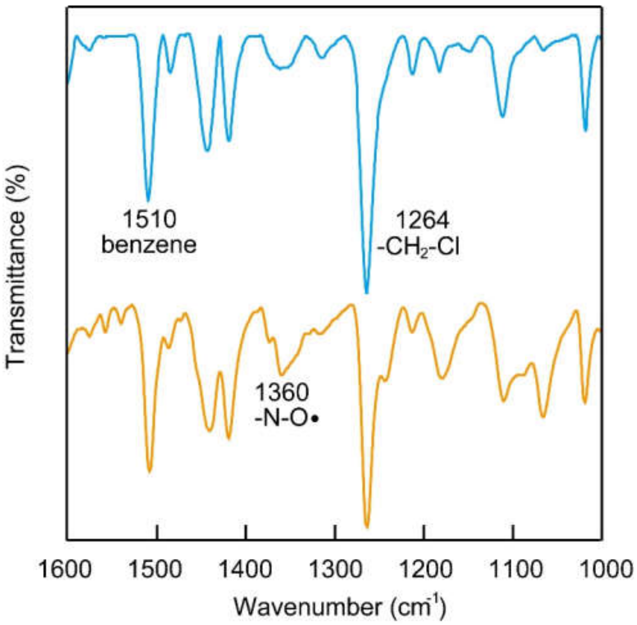

Figure S5. The FT-IR results of Cl−Monolith−1 (top) and Monolith−1 (bottom).

3. Flow behavior analysis of polymer monolith with tracers

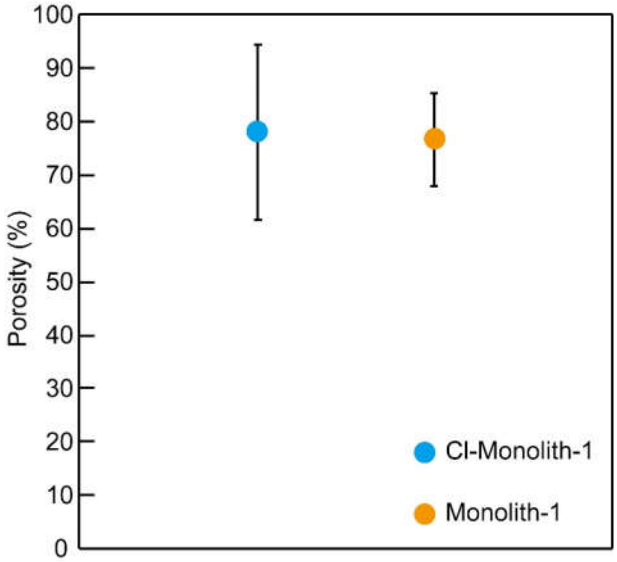

Figure S6. Porosity measurements in wetting states with benzene tracer in CH_2Cl_2/^{t}BuOH (1:1, *v*/*v*).

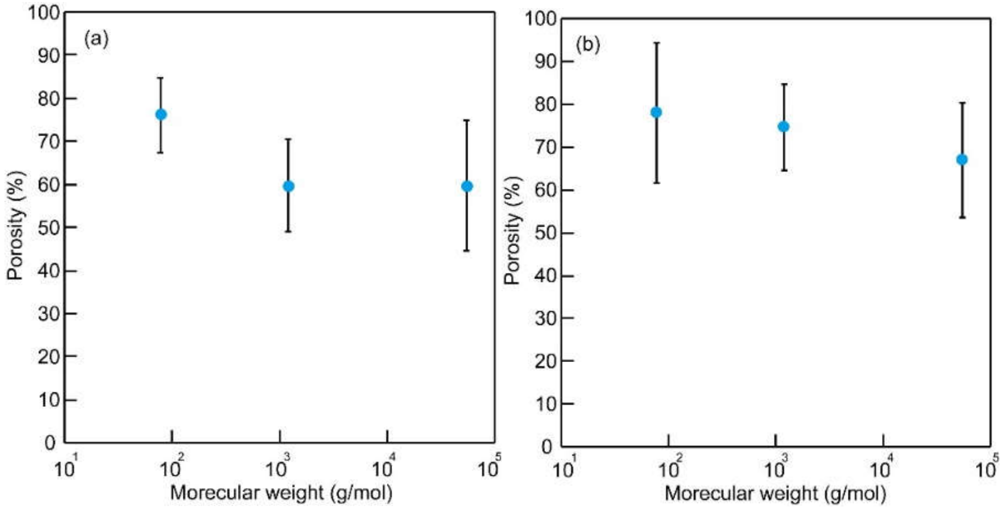

Figure S7. Porosity measurements in wetting states with benzene and polystyrene tracer in CH_2Cl_2/^{t}BuOH (1:1, *v*/*v*): (a) Monolith−1 and (b) Cl−Monolith−1.

4. Flow oxidation reaction with Monolith-1

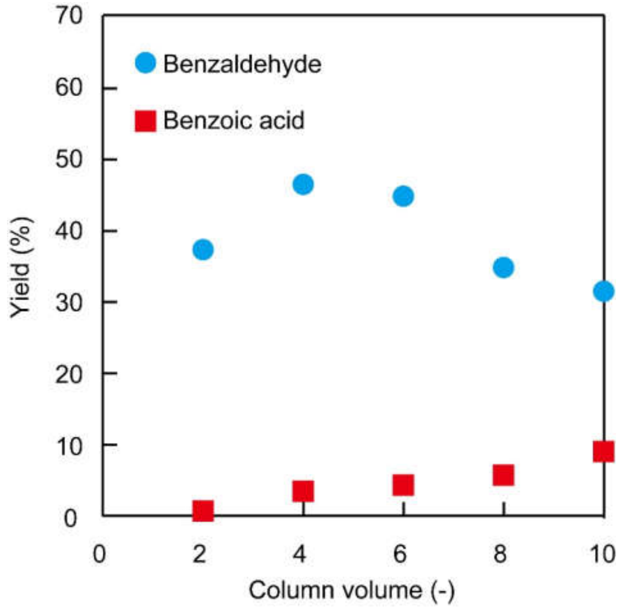

Figure S8. Results of flow oxidation of benzyl alcohol by Monolith−1 at a residence time of 2 min.

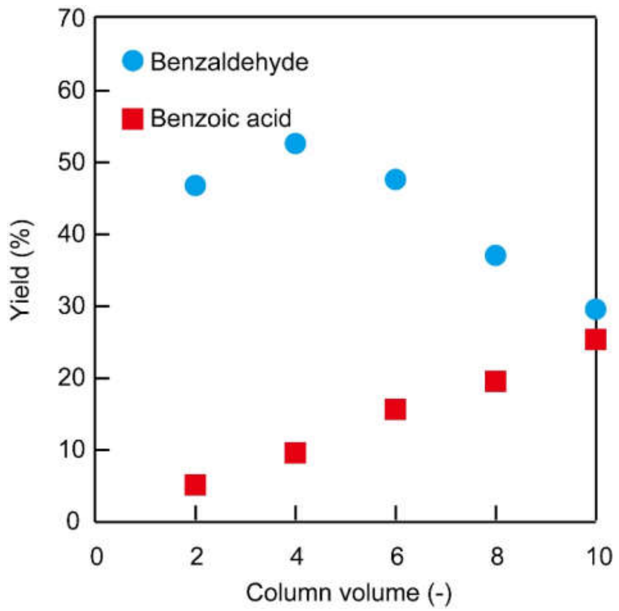

Figure S9. Results of flow oxidation of benzyl alcohol by Monolith−1 at a residence time of 8 min.

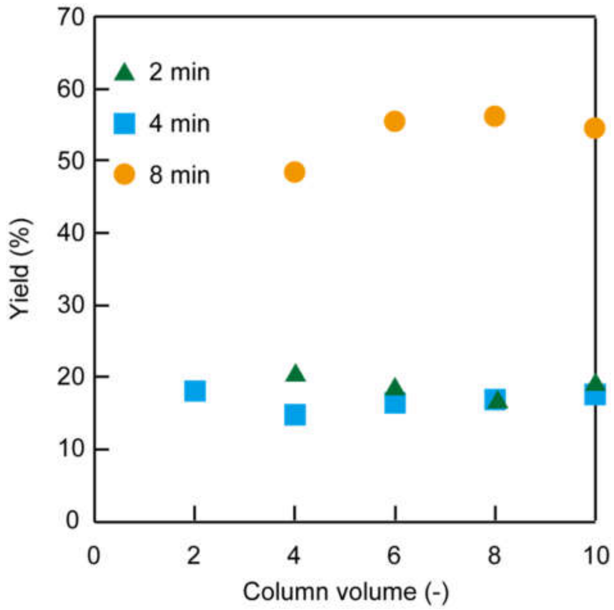

Figure S10. Results of flow oxidation of 2-phenylethanol by Monolith−1 at residence times of 2, 4, and 8 min.

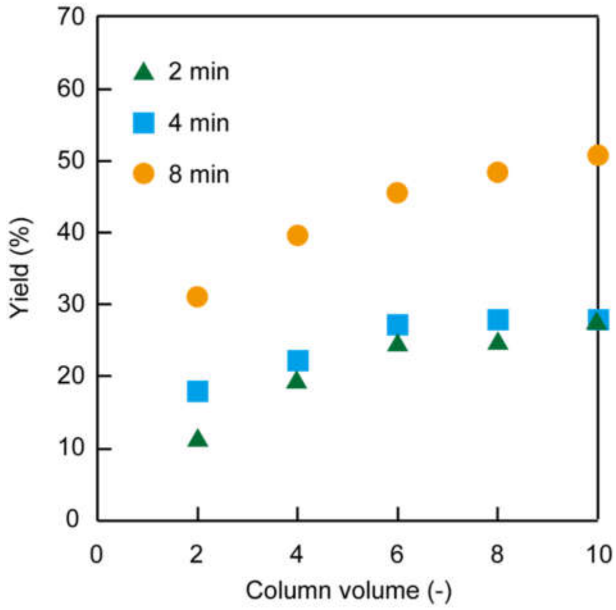

Figure S11. Results of flow oxidation of (+/-)-1-phenylethanol by Monolith−1 at residence times of 2, 4, and 8 min.
